# 14179 Retrospective Case Studies using the TSBM to Evaluate Translation Research Progress

**DOI:** 10.1017/cts.2021.578

**Published:** 2021-03-30

**Authors:** Ingrid Philibert, Jolene Rohde, LaKaija Johnson

**Affiliations:** 1 Great Plains IDeA CTR, University of Nebraska Medical Center; 2 Great Plains IDeA CTR; 3 Assistant Director of Team Science, ICTR, University of Wisconsin

## Abstract

ABSTRACT IMPACT: This effort will ultimately improve both human and community health and translational science by showing the impact of CTR services on different types of projects that meet overall CTR missions and aims. OBJECTIVES/GOALS: CTRs seek to advance translational research to generate clinical, healthcare delivery, policy and community benefits. We conducted retrospective case studies for selected funded Pilot Projects for the Great Plains IDeA-CTR, focusing on facilitators and barriers to research translation and contrasting community-engaged and other proposals. METHODS/STUDY POPULATION: We analyzed 8 CTR-funded projects (4 community-engaged (CE) projects and 4 other pilot awards) focusing on outcome domains of the Translational Science Benefits Model (TSBM): Clinical, Economic, Policy and Community Benefits as endpoints of successful research translation. We adapted an existing TSBM case study template for use with data required by NIH/NGIMS to map progress toward one or more TSBM outcomes. Using email, we posed three brief open-ended questions to investigators: 1) challenges/ barriers for the project; 2) how the CTR helped move research along and (how it could have moved it further); and 3) how research is progressing and how it could progress further. RESULTS/ANTICIPATED RESULTS: All investigators reported the CTR advanced their project. Non-CE projects appeared to have a more straightforward trajectory, with 2 investigators reporting no challenges and 2 reporting solely institution-internal ones. In contrast, the 4 CE projects reported both benefit from the engagement of the CTR (most prominently the efforts of the community advisory board (CAB) and community liaisons). Yet, they also reported some challenges beyond the CTR’s ability to address, including delays in securing community buy-in and community buy-in of the investigator’s research approach. Some barriers appeared beyond the CTR’s current immediate ability to provide support to advance the project. DISCUSSION/SIGNIFICANCE OF FINDINGS: Findings contribute to efficient approaches for retrospective case studies and emerging information on challenges and opportunities for CE projects. The study will help identify: 1) intermediate milestones and timelines for different projects; 2) advance data for TBSM endpoints; and 3) CTR activities that leverage the translational process.

